# The relationship between thyroid dysfunction and nephrotic syndrome: a clinicopathological study

**DOI:** 10.1038/s41598-019-42905-4

**Published:** 2019-04-23

**Authors:** Ling-Zhi Li, Yao Hu, Shuang-Lan Ai, Lu Cheng, Jing Liu, Emily Morris, Yi Li, Shen-Ju Gou, Ping Fu

**Affiliations:** 1 Renal Division, Department of Medicine, West China Hospital of Sichuan University, Kidney Research Institute, Chengdu, 610041 China; 2 Renal Division, Department of Medicine, Affiliated Hospital of Chengdu University, Chengdu, 610081 China; 3 Department of Biostatistics, School of Public Health, University of Michigan, Ann Arbor, MI United States of America

**Keywords:** Thyroid diseases, Kidney diseases

## Abstract

Abnormalities of thyroid function are common in patients with nephrotic syndrome (NS). However, a limited number of studies have reported on the association between clinicopathologic features and thyroid dysfunction in patients with NS. We retrospectively studied 317 patients who had been definitively diagnosed with NS. The NS patients with thyroid dysfunction showed higher urine protein, creatinine and lipid levels and lower albumin and hemoglobin than those with normal thyroid function, with no significant differences of pathological types. After dividing thyroid dysfunction groups into five subgroups, interestingly, membranous nephropathy was the most common pathologic type, both in normal thyroid group and in subclinical hypothyroidism group (40.4% and 46.7%, respectively), followed by minimal change disease (28.1% and 21.7%, respectively); while in the hypothyroid, low T3, and low T3T4 groups minimal change disease is now the leading type (48.8%, 33.3% and 38.6%, respectively). High levels of urinary protein, creatinine, cholesterol, and platelets were independent risk factors predicting thyroid dysfunction, while higher albumin and hemoglobin were protective factors. We demonstrated that the type of renal pathology was different among NS patients in different thyroid dysfunction subgroups. Interpretation of the interactions between thyroid and renal function is a challenge for clinicians involved in the treatment of patients with NS.

## Introduction

Nephrotic syndrome (NS) is one of the most common glomerular diseases and is defined by edema, substantial proteinuria (>3.5 g/24 hours), hypoalbuminemia (<30 g/L) and hyperlipidemia. It is often associated with thromboembolism and an increased risk of infection^1^. Endocrine abnormalities are common in NS patients. As a primary endocrine organ, the thyroid plays an important role in kidney growth and function^2,3^. Kidneys in return play a crucial role in metabolism and elimination of thyroid hormone (TH)^4^. A variety of altered thyroid hormone levels and metabolisms such as hypothyroidism and subclinical hypothyroidism (SCH) have been reported in NS and CKD patients^5–7^. Recently, much interest has been focused on euthyroid sick syndrome (ESS), which is characterized by decreased serum FT3 and/or FT4 levels and no significant increase in TSH^8^. However, the data related to different types of thyroid dysfunction in NS, especially the relationship between thyroid function and pathological characteristics of NS patients, are scarce. This study was to evaluate the thyroid hormone profile in patients with NS, to identify clinical predictors of thyroid dysfunction in patients with NS, and to analyze the associations of pathological characteristics with the thyroid function among patients with NS.

## Results

### Baseline demographics and clinical characteristics of the enrolled patients

Initially, 348 NS patients were enrolled from January 2013 to December 2016. After excluding patients with primary thyroid disease (including two patients with hyperthyroidism) and patients without a renal biopsy or data on thyroid function, 317 patients remained in the study. Among these patients, we found that 140 (44.16%) underwent the testing after steroid therapy. The baseline demographics and clinical characteristics of the enrolled patients were summarized in Table 1. We first divided NS patients into the normal thyroid function group (n = 57) and the thyroid dysfunction group (n = 260). The mean age, sex, and duration of NS did not differ between the two groups. However, PRO was significantly higher, and serum ALB was significantly lower in NS patients with thyroid dysfunction compared to those with normal thyroid function (8.27 ± 6.56 g/24 h vs. 4.03 ± 2.45 g/24 h, P < 0.001; 22.08 ± 5.88 g/L vs. 28.68 ± 6.19 g/L, P < 0.001; respectively). Blood lipid parameters at presentation showed that the levels of TC and LDL were significantly higher in the thyroid dysfunction group than those in the normal thyroid function group (8.48 ± 3.23 g/L vs. 7.15 ± 2.24 g/L, P < 0.001; 5.54 ± 2.78 g/L vs. 4.54 ± 1.93 g/L, P = 0.002; respectively). Additionally, the levels of serum creatinine were significantly higher in patients with thyroid dysfunction than those with normal thyroid function (70.00 [55.00, 104.95] umol/L vs. 55.00 [61.00, 75.5] umol/L, P = 0.027). The level of hemoglobin was significantly lower in patients with thyroid dysfunction than those with normal thyroid function (132.24 ± 25.17 g/L vs. 143.14 ± 23.55 g/L, P = 0.003).

**Table 1:** Comparisons of clinical features and laboratory findings between the normal thyroid group and the thyroid dysfunction group.

	Normal thyroid (n = 57)	Thyroid dysfunction (n = 260)	P
**Clinical characteristics**			
Age (Year)	41.3 ± 14.33	40.65 ± 16.16	0.779
Male (%)	31(54.40%)	139(53.60%)	0.517
Duration of NS (Month)	3.00 [1.00, 8.00]	6.00 [1.00, 10.00]	0.098
HB (g/L)	143.14 ± 23.55	132.24 ± 25.17	0.003
WBC (10^9^/L)	7.36 ± 2.53	8.02 ± 3.41	0.099
PLT (10^9^/L)	202.23 ± 72.02	232.79 ± 86.77	0.014
UA (umol/L)	354.36 ± 87.87	361.52 ± 102.29	0.637
PRO (g/24 h)	4.03 ± 2.45	8.27 ± 6.56	<0.001
PCR (g/mmol Cr)	0.47 ± 0.4	0.9 ± 0.6	<0.001
ALB (g/L)	28.68 ± 6.19	22.08 ± 5.88	<0.001
SCR (umol/L)	55.00 [61.00, 75.5]	70.00 [55.00, 104.95]	0.027
TG (mmol/L)	2.03 ± 1.06	2.46 ± 1.6	0.055
TC (mmol/L)	7.15 ± 2.24	8.48 ± 3.23	<0.001
LDL (mmol/L)	4.54 ± 1.93	5.54 ± 2.78	0.002
FT3 (pmol/L)	4.49 ± 0.64	3.17 ± 1.39	<0.001
TT3 (nmol/L)	1.64 ± 0.26	1.2 ± 0.43	<0.001
FT4 (pmol/L)	15.8 ± 2.18	12.81 ± 3.42	<0.001
TT4 (nmol/L)	87.32 ± 12.8	66.07 ± 25.38	<0.001
TSH (mU/L)	4.57 [2.60, 6.81]	3.00 [2.02, 3.60]	<0.001
RT3 (nmol/L)	0.36 ± 0.14	0.3 ± 0.15	0.215
**Coexisting diseases** (**%**)			
Hypertension	17.50%	20.20%	0.653
Diabetes	1.80%	5.70%	0.365
Pulmonary infection	7.00%	15.20%	0.157
AKI	1.80%	4.20%	0.624
CKD	1.80%	8.00%	0.163
**Renal biopsy** (**%**)			
MN	40.4%	30.7%	0.339
MCD	28.1%	36.2%	
IgAN	10.5%	7.8%	
MPGN	3.5%	1.9%	
MsPGN	0.00%	3.50%	
FSGS	3.5%	3.5%	
Secondary NS	14.0%	16.3%	

Data are expressed as mean ± standard deviation, median and interquartile range, or percent frequency, as appropriate.

*MN* membranous nephropathy, *MCD* minimal-change disease, *FSGS* focal segmental glomerulosclerosis, *MPGN* membrane proliferative glomerulonephritis, *MsPGN* mesangial proliferative glomerulonephritis, *secondary NS* secondary reasons of nephrotic syndrome.

### The pathological characteristics of enrolled patients

Pathological analyses revealed that membranous nephropathy (MN) and minimal-change disease (MCD) were the main pathological types both in the normal thyroid group (40.4% and 28.1%, respectively) and in the thyroid dysfunction group (30.7% and 36.2%, respectively) (Table 1). No significant differences of the pathological compositions were reported (P = 0.339).

### Comparison of clinical characteristics among the normal thyroid function group and thyroid dysfunction groups

The comparison results of baseline clinical characteristics among the normal thyroid function group and different thyroid dysfunction groups were listed in Table 2. Among the included 260 NS patients with thyroid dysfunction, hypothyroidism was most common (n = 83, 31.9%), followed by Low T3 (n = 66, 25.4%), subclinical hypothyroidism (n = 61, 23.5%), Low T3T4 (n = 45, 17.3%) and Low T4 (n = 5, 1.9%). The mean age, sex, and duration of NS did not differ among these subgroups. Compared with the normal thyroid group, hypothyroidism and low T3T4 groups had higher levels of unitary protein (4.03 ± 2.45 g/24 h vs. 9.71 ± 7.42 g/24 h, P < 0.05; 4.03 ± 2.45 g/24 h vs. 10.85 ± 8.62 g/24 h, P < 0.05; respectively). Additionally, hypothyroidism and low T3T4 groups had lower levels of serum albumin than that of the normal thyroid function group (19.01 ± 4.56 g/L vs. 28.68 ± 6.19 g/L, P < 0.05; 19.48 ± 3.99 g/L vs. 28.68 ± 6.19 g/L, P < 0.05; respectively). The levels of SCR and TC were obviously higher in the low T3 group compared to those in the normal thyroid group (73.80 [52.60, 119.00] vs. 61.50 [55.00, 75.00] umol/L, P < 0.05; 7.19 ± 2.98 vs. 7.15 ± 2.24 mmol/L, P < 0.05; respectively). Similarly, the levels of SCR and TC were also higher in the low T3T4 group than those in the normal thyroid group (79.90 [59.35, 138.30] vs. 61.50 [55.00, 75.00] umol/L, P < 0.05; 9.44 ± 3.66 vs. 7.15 ± 2.24 mmol/L, P < 0.05; respectively). The hemoglobin level was significantly lower in the low T3 group than that in the normal thyroid group (123.95 ± 26.87 g/L vs. 143.14 ± 23.55 g/L, P < 0.05). The subgroups of the thyroid dysfunction group, except for subclinical hypothyroidism and low T4, had higher percentages of pulmonary infection than that of NS patients with normal thyroid function (P = 0.04).

**Table 2:** Comparisons of clinical features and laboratory findings by different thyroid dysfunction identification.

	Normal Thyroid	Subclinical hypothyroidism	Hypothyroidism	Euthyroid sick syndrome			P
				Low T3	Low T4	Low T3T4	
**Clinical characteristics**							
N	57	61	83	66	5	45	—
Age (Year)	41.3 ± 14.33	41.72 ± 15.44	38.24 ± 16.79	42.92 ± 15.9	41.4 ± 16.3	40.33 ± 16.65	0.604
Male (%)	54.4%	62.3%	49.4%	50.0%	60.0%	53.3%	0.714
Duration of NS (Month)	6.00 [1.00, 10.00]	4.00 [1.00, 7.00]	2.00 [1.00, 8.00]	3.00 [1.00, 8.00]	3.00 [2.00, 6.00]	2.00 [1.00, 7.75]	0.210
PRO (g/24 h)	4.03 ± 2.45	6.51 ± 4.33	9.71 ± 7.42*	6.71 ± 4.9	7.26 ± 5.22	10.85 ± 8.62*	<0.001
PCR (g/mmol Cr)	0.47 ± 0.4	0.79 ± 0.51	1.01 ± 0.58*	0.76 ± 0.53	0.76 ± 0.37	1.02 ± 0.8*	<0.001
ALB (g/L)	28.68 ± 6.19	24.19 ± 5.65*	19.01 ± 4.56*	25.52 ± 6.11*	22.88 ± 4.96	19.48 ± 3.99*	<0.001
SCR (umol/L)	61.50 [55.00, 75.00]	68.00 [56.00, 84.30]	69.00 [53.00, 94.70]	73.80 [52.60, 119.00]*	54.00 [53.00, 67.00]	79.90 [59.35, 138.30]*	<0.001
TG (mmol/L)	354.36 ± 87.87	366.4 ± 101.97	343.16 ± 81.35	372.28 ± 123.63	346.66 ± 97.66	373.26 ± 105.08	0.525
TC (mmol/L)	2.03 ± 1.06	2.5 ± 1.51	2.53 ± 1.8	2.14 ± 1.18	1.83 ± 0.63	2.83 ± 1.88*	0.062
LDL (mmol/L)	7.15 ± 2.24	8.14 ± 2.96	9.37 ± 3.11	7.19 ± 2.98*	6.86 ± 1.84	9.44 ± 3.66*	<0.001
FT3 (pmol/L)	4.54 ± 1.93	5.3 ± 2.5	6.27 ± 2.69*	4.44 ± 2.41	4.33 ± 1.2	6.31 ± 3.38*	<0.001
TT3 (nmol/L)	4.49 ± 0.64	4.31 ± 1	2.76 ± 0.71*	3.11 ± 1.9*	3.95 ± 0.47	2.28 ± 0.89*	<0.001
FT4 (pmol/L)	1.64 ± 0.26	1.61 ± 0.43	1.08 ± 0.27	1.14 ± 0.35	2.04 ± 0.2	0.93 ± 0.36	<0.001
TT4 (nmol/L)	15.8 ± 2.18	14.7 ± 3.02	11.05 ± 2.68*	14.61 ± 2.32*	10.74 ± 1.07*	10.34 ± 1.42*	<0.001
TSH (mU/L)	87.32 ± 12.8	84.87 ± 19.35	49.72 ± 16.71	86.53 ± 24.48	83.51 ± 1.2	48.85 ± 9.67	<0.001
RT3 (nmol/L)	2.98 [2.05, 3.60]	6.25 [5.1, 7.16]	6.98 [5.54, 9.04]	2.6 [1.50, 3.15]	3.23 [2.00, 3.27]	2.45 [1.67, 3.09]	<0.001
PRO(g/24 h)	0.36 ± 0.14	0.33 ± 0.12	0.25 ± 0.15	0.32 ± 0.11	0.28 ± 0	0.36 ± 0.16	0.214
HB (g/L)	143.14 ± 23.55	134.44 ± 26.16	135.7 ± 22.89	123.95 ± 26.87*	134.6 ± 11.44	133.8 ± 24.84	0.002
WBC (10^9/L)	7.36 ± 2.53	7.87 ± 2.92	7.47 ± 2.66	8.12 ± 3.72	6.19 ± 3	9.13 ± 4.25*	0.045
PLT (10^9/L)	202.23 ± 72.02	237.98 ± 97.93	241.43 ± 76.58*	213.26 ± 90.17	214.6 ± 29.06	241.98 ± 88.11	0.043
**Coexisting diseases** (**%**)							
Hypertension	17.5%	26.2%	10.8%	27.3%	20.0%	17.8%	0.120
Diabetes	1.8%	6.6%	1.2%	10.6%	0.0%	6.7%	0.086
Pulmonary infection	7.0%	6.6%	16.9%*	15.2%*	0.0%	24.4%*	0.040
AKI	1.8%	0.0%	4.8%	4.5%	0.0%	8.9%	0.127
CKD	1.8%	4.9%	3.6%	12.1%	0.0%	13.3%	0.058
**Renal biopsy** (**%**)							
MCD	28.1%	21.7%	48.8%	33.3%	20.0%	38.6%	<0.001
MPGN	3.5%	1.7%	1.2%	0.0%	0.0%	6.8%	
MsPGN	0.0%	1.7%	2.4%	4.8%	0.0%	6.8%	
MN	40.4%	46.7%	22.0%	23.8%	0.0%	27.3%	
FSGS	3.5%	5.0%	1.2%	3.2%	80.0%	6.8%	
IgAN	10.5%	10.0%	6.1%	11.1%	0.0%	4.5%	
Secondary NS	10.9%	11.3%	14.9%	22.0%	0.0%	8.6%	

Data are expressed as mean ± standard deviation, median and interquartile range, or percent frequency, as appropriate.

*MN* membranous nephropathy, *MCD* minimal-change disease, *FSGS* focal segmental glomerulosclerosis, *MPGN* membrane proliferative glomerulonephritis, *MsPGN* mesangial proliferative glomerulonephritis, *secondary* secondary reasons of nephrotic syndrome.

*P < 0.05, Comparison versus normal thyroid group.

### The pathological characteristics of patients with normal thyroid function and thyroid dysfunction

The renal pathologic types among the subgroups were also listed in Table 2, which showed significant differences among the six groups (P < 0.001). Among the patients with normal thyroid function and those with subclinical hypothyroidism, MN was the most common pathologic type (40.4%), followed by MCD (28.1%). The hypothyroidism, low T3, and low T3T4 groups showed a contrasting result, in which MCD was now the leading pathologic type, consisting of 48.8%, 33.3%, and 38.6% respectively. For the low T4 group, 80% of the patients were FSGS.

After analyzing the patients without pretreatment of steroid separately, it was found that the compositions of pathological types in different thyroid dysfunction subgroups were similar to that of all NS patients, which suggested the differences of pathological types in different thyroid dysfunction subgroups existed regardless of the steroid therapy (Supplementary Table S1). Additionally, we have also analyzed the clinical features and laboratory findings in different thyroid dysfunction identification according to albumin level (Supplementary Table S2). After adjusted by albumin levels, we found that in subgroups of patients with albumin between 17 g/L and 20 g/L, the structure of renal pathological types was still significantly different among thyroid dysfunction subgroups.

### The thyroid function characteristics according to different pathological types

The thyroid related laboratory findings with pathological classifications were summarized in Table 3. The level of FT3 was higher in the MN group compared with that of the MCD group (3.91 ± 1.75 vs 3.09 ± 1.06 umol/L, P = 0.01). The proportion of different thyroid function subgroups also differed in patients with different renal pathological types (P = 0.028). The most common thyroid dysfunction type in MCD and MN was hypothyroidism (37.04%) and subclinical hypothyroidism (28%), respectively. However, in membrane proliferative glomerulonephritis (MPGN) and FSGS groups, low T3T4 was the most common type (42.86% and 27.27%, respectively). In the mesangial proliferative glomerulonephritis (MsPGN) group, low T3 and low T3T4 both consisted of 33.33%. The other related clinical characteristics were summarized in Supplementary Table S3.

**Table 3:** Thyroid function according to different pathologic classifications.

	MCD	MPGN	MsPGN	MN	FSGS	IgAN	Secondary	P^#^
N	110	7	9	98	15	27	51	—
Age (Year)	34.29 ± 16.17	38.86 ± 8.88	40.89 ± 15.09	47.85 ± 13.69*	41.64 ± 17.48	38.04 ± 15.31	42.8 ± 14.88	<0.001
Male (%)	55.5%	71.4%	33.3%	55.2%	45.5%	34.6%	60.8%	0.250
FT3 (pmol/L)	3.09 ± 1.06	3.07 ± 0.91	2.95 ± 0.97	3.91 ± 1.75*	3.66 ± 1.63	3.36 ± 1.17	3.24 ± 1.02	0.001
TT3 (nmol/L)	1.16 ± 0.49	0.53 ± 0.1	1.09 ± 0.39	1.49 ± 0.43*	1.27 ± 0.1	1.31 ± 0.17	1.45 ± 0.41	0.058
FT4 (pmol/L)	12.68 ± 3.22	12.92 ± 2.25	13.47 ± 4	13.68 ± 3.53*	14.51 ± 4.55	14.09 ± 3.67	13.59 ± 3.34	0.256
TT4 (nmol/L)	59.33 ± 21.24	44.61 ± 0.3	78.79 ± 22.78*	73.99 ± 22.61*	106.5 ± 0.2*	91.09 ± 14.05*	79.85 ± 29.38*	0.014
TSH (mU/L)	5.42 ± 5.29	3.31 ± 1.64	3.7 ± 2.59	4.36 ± 2.81	4.33 ± 3.44	5.38 ± 3.94	6.45 ± 13.63	0.571
RT3 (nmol/L)	0.27 ± 0.13	0.33 ± 0.1	0.43 ± 0.06	0.35 ± 0.19	0.29 ± 0.15	0.3 ± 0.1	0.3 ± 0.15	0.403
Thyroid subgroups								0.028
Normal Thyroid (%)	14.81%	28.57%	0.00%	23.00%	18.18%	23.08%	16.00%	
Subclinical Hypothyroidism (%)	12.04%	14.29%	11.11%	28.00%	27.27%	23.08%	16.00%	
Hypothyroidism (%)	37.04%	14.29%	22.22%	18.00%	9.09%	19.23%	30.00%	
Low T3 (%)	19.44%	0.00%	33.33%	15.00%	18.18%	26.92%	30.00%	
Low T4 (%)	0.93%	0.00%	0.00%	4.00%	0.00%	0.00%	0.00%	
Low T3T4 (%)	15.74%	42.86%	33.33%	12.00%	27.27%	7.69%	8.00%	

Data are expressed as mean ± standard deviation (SD), median and interquartile range (IQR), or percent frequency, as appropriate.

*MN* membranous nephropathy, *MCD* minimal-change disease, *FSGS* focal segmental glomerulosclerosis, *MPGN* membrane proliferative glomerulonephritis, *MsPGN* mesangial proliferative glomerulonephritis, *secondary* secondary reasons of nephrotic syndrome.

^#^Comparison among groups. *P < 0.05, Comparison versus MCD group.

### Correlations between clinical parameters and thyroid dysfunction

Clinical predictors of thyroid dysfunction were assessed by multiple logistic regression models. The correlation analysis of these parameters was reported in the Supplementary Table S4. High levels of PRO, SCR, TC, and PLT were independent risk factors for predicting thyroid dysfunction, while a high level of ALB and Hb were two independent protective factors (Table 4). However, renal pathological types were not independent risk or protective factors for predicting thyroid dysfunction.

**Table 4:** Multiple regression model of variables predicting thyroid dysfunction in NS patients.

	B	SE	OR	95% CI	P
Sex (Male/female)	−0.233	0.469	0.792	0.32–1.99	0.619
Age (Year)	−0.017	0.013	0.983	0.958–1.008	0.181
Hypertension	0.298	0.511	1.347	0.5–3.67	0.560
Diabetes	2.086	1.372	8.056	0.55–118.55	0.128
Pneumonia	0.692	0.781	1.997	0.43–9.22	0.375
PRO (g/24 h)	0.128	0.056	1.137	1.02–1.27	0.021
ALB (g/L)	−0.149	0.038	0.862	0.8–0.93	0.000
SCR (umol/L)	0.012	0.006	1.013	1–1.03	0.050
TC (mmol/L)	0.611	0.252	1.843	1.12–3.02	0.015
HB (g/L)	−0.018	0.008	0.983	0.97–1	0.022
PLT (10^9^/L)	0.006	0.003	1.006	1–1.01	0.020
WBC (10^9^/L)	0.088	0.077	1.092	0.94–1.27	0.250
Pathological types	0.052	0.475	1.053	0.42–2.67	0.913

*PRO* 24 h urinary protein, *ALB* albumin, *SCR* serum creatinine, *TC* total cholesterol, *HB* hemoglobin, *WBC* white blood cell count, *PLT* platelet.

## Discussion

NS patients with abnormal thyroid function are very common. Generally, NS upsets thyroid function through low circulating thyroid hormone concentration, insufficient binding to carrier proteins or altered iodine storage in the thyroid gland^9–11^. In the present study, 260 of the 317 NS patients (82.0%) showed abnormal thyroid function, and 116 NS patients (36.6%) had euthyroid sick syndrome.

In our study NS patients with thyroid dysfunction expressed higher levels of urinary protein, especially for hypothyroidism and low T3T4 subgroups, compared to NS patients with normal thyroid function. The loss of a large number of plasma proteins from urine is the most fundamental pathophysiological change of NS. For NS patients, the more proteinuria discharged, the more thyroid binding globulin lost and the higher the metabolic rate of thyroid hormone^12^, which might contribute to the possibility of thyroid dysfunction. Additionally, hypoalbuminemia may be another cause of the decrease of FT3 and FT4. In our study, the level of serum albumin was lower in NS patients with thyroid dysfunction compared with NS patients with normal thyroid function, and the high level of albumin was an independent protective factor of thyroid function. Albumin serves as a buffer protein of TH in the plasma, when its level is considerably low the TH vector is reduced and no sufficient TH can be dissociated from the protein hormone complex. Furthermore, the massive proteinuria and hypoproteinemia might lead to gastrointestinal mucous membrane edema to influence protein intake, resulting in the deficiency of TH synthetic materials and reduction of TH production.

The differences of renal pathologic types might be another contributing reason. Our study did not detect statistical differences in the constituent ratio of the pathological types between the normal thyroid function group and the thyroid dysfunction group. Interestingly, after further subdivision of the thyroid dysfunction group, the TH level was related to different types of renal pathology. In our study, MN was the most prevalent primary glomerulopathy diagnosed in the cohort of NS patients with normal thyroid function and patients with subclinical hypothyroidism. However, hypothyroidism and euthyroid sick syndrome groups (low T3 and low T3T4 groups) showed a contrasting renal pathologic structure from the normal thyroid function group, where MCD was the most common renal pathologic type (consisted of 48.8%, 33.3%, and 38.6%, respectively). In previous reports, MCD accounted for approximately 20% of NS adults^13^. The reported proportion was evidently lower than that of NS patients with thyroid dysfunction in the present study, which emphasized the differences of renal pathological structure in this population. Although the differences of pathological types existed in different thyroid dysfunction subgroups, the multivariate logistic regression showed that pathological types were not independent predictors. It was possible that the main causes of thyroid dysfunction were changes of clinical manifestations: albuminuria and serum albumin levels from our multivariate logistic regression analysis and previous studies^3,12,14^. But the roots of different clinical characteristics defined the differences of pathologic types and pathologic severity^1^. Since there were no uniform criteria for the assessment of pathologic severity in different pathologic types and the pathologic severity was not evaluated by the present study, it could not rule out the association of pathology with thyroid dysfunction in patients with NS. The present study still contributes to the total body of knowledge concerning the patterns of renal pathology among NS individuals with thyroid dysfunction and provides more insight for detecting the causes of thyroid dysfunction in NS patients.

FT3 and FT4, as the active part of TH, play biological roles in tissue metabolism, and their decline will affect the function of all systems in the body, causing hyperlipidemia, anemia, renal function deficiency, and so on. The level of plasma lipid was higher in NS patients with thyroid dysfunction in our study, which was consistent with previous studies that hypothyroidism could lead to a further increase in hyperlipidemia and thus increase the risk of thromboembolism and glomerulosclerosis^15^. Besides hyperlipidemia, in this study the level of HB was much lower in the low T3 group compared to NS patients with normal thyroid function (P < 0.05). Investigation of a relationship between thyroid dysfunction and anemia should be considered. Several reports have shown that hypothyroidism may contribute to erythropoietin resistance in chronic hemodialysis patients and that early thyroxine administration can improve anemia significantly^16–18^. The effects of thyroid hormones on hematopoiesis have been documented, such as an increase in production of erythropoietin or hematopoietic factors by nonerythroid cells^19^ and regulation of the intestinal absorption of folic acid and vitamin B12^20^. Taken together, these studies imply that the specific metabolic environment in NS may induce anemia by influencing thyroid hormones.

In conclusion, thyroid dysfunction is common in patients with nephrotic syndrome. The type of renal pathology was different in thyroid dysfunction subgroups in patients with nephrotic syndrome. Interpretation of the interactions between thyroid and renal function is a challenge for clinicians involved in the treatment of patients with nephrotic syndrome. As a consequence, appropriate cooperation between endocrinologists and nephrologists is needed to properly interpret the changes in thyroid and kidney function in patients with nephrotic syndrome.

## Methods

### Patients selection and clinical data collection

Upon reviewing medical records, we enrolled patients aged 18 years or older who were diagnosed with NS at the West China Hospital of Sichuan University between January 2013 and December 2016. The inclusion criteria of NS required the following features: (1) generalized edema; (2) urine protein excretion ⩾3.5 g/24 h or ⩾3,500 mg/g measured by the spot urine protein-to-creatinine ratio (PCR), and (3) serum albumin level less than 30 g/l. The study excluded (1) patients aged less than 18 years, and (2) patients who lacked thyroid related laboratory tests or renal biopsy results.

Initially, we recorded demographic information (name, sex, age, etc.) and the presence of coexisting diseases (hypertension and diabetes, etc.). Fasting blood samples were collected from the peripheral vein in the morning during the first three days of hospitalization and analyzed for indicators of thyroid function (serum total T3 (TT3), free T3 (FT3), total T4 (TT4), free T4 (FT4), thyroid stimulating hormone (TSH) and reverse triiodothyronine (RT3)), indicators of renal function (serum creatinine, SCR), hemoglobin (HB), white blood cell (WBC) count, platelet (PLT) count, and blood lipids (triglyceride(TG), cholesterol(TC), low density lipoprotein(LDL)). Routine urine samples were collected, and 24 h urinary protein (PRO) and urine protein creatinine ratio (PCR) were tested. Renal biopsy was performed and the specimens were examined by light microscopy, immunofluorescence microscopy, and electron microscopy. The present study was approved by the ethics committee of West China Hospital of Sichuan University and all included patients gave their informed consent. All the procedures followed the Declaration of Helsinki principles.

### Thyroid dysfunction identification and pathological classification

Subclinical hypothyroidism (SCH) is defined as an elevation in TSH levels despite normal serum levels of free thyroxine^21^. Hypothyroidism is characterized by low FT4 and FT3 with elevated TSH. Sick euthyroid syndrome, which is also known as non-thyroidal illness syndrome, could be categorized into three main groups: low T3, low T4, and low T3T4. The low T3 is characterized by a low serum level of FT3 accompanied by normal FT4 and normal TSH levels^22^. The low T4 is defined as low serum FT4 level with normal FT3 level and TSH level. A normal serum TSH level with low serum FT3 and FT4 level is defined as low T3T4.

The renal specimens obtained from the patients with NS were classified according to 1990 WHO classification criteria. For identification of the secondary causes of NS, initial laboratory data were also reviewed, including (1) tests for serum autoantibodies such as antinuclear antibody, anti-double-stranded DNA antibody, anti-neutrophil cytoplasmic antibody, anti-Smith, rheumatoid factor and complement; (2) tests for hepatitis B, hepatitis C, human immunodeficiency virus and syphilis infection; (3) serum protein electrophoresis and immunofixation electrophoresis.

### Statistical analysis

Statistical analyses were performed using SPSS Statistics for Windows Version 22.0. (Chicago, IL: SPSS Inc.). Continuous data with normal distribution were presented as means ± standard deviation (SD). Non-normal data were presented as the median and quartile. Normal data were compared using Student’s t-test between two groups or ANOVA testing among more groups, while non-normal data were compared by rank-sum tests. Categorical data were compared using the chi-square test or Fisher’s exact test. Associations between clinical parameters were assessed by Spearman correlations. Multivariate analysis of risk factors affecting thyroid dysfunction was performed using logistic regression models. The estimated standard error of the coefficient estimate was used to establish confidence intervals (CI) of the estimated odds ratio (OR). Two-sided P values < 0.05 were considered statistically significant.

## Supplementary information

supplementary information
