# Supplementary material for: The relationship between thyroid dysfunction and nephrotic syndrome: a clinicopathological study
Source: Sci Rep. 2019 Apr 23;9:6421. doi: 10.1038/s41598-019-42905-4 (PMC6478922; doi:10.1038/s41598-019-42905-4)
Supplement: Supplementary file 1 — supplementary information [file 41598_2019_42905_MOESM1_ESM.docx]

**Title The relationship between thyroid dysfunction and nephrotic syndrome: a clinicopathological study**

**Running Title Thyroid dysfunction and NS**

**Authors Ling-Zhi Li*^1^, Yao Hu*^1,2^, Shuang-Lan Ai^1^, Lu Cheng^1^, Jing Liu^1^, Emily Morris^3^, Yi Li^3^, Shen-Ju Gou^1,^ Ping Fu^1^**

***contributed equally to this paper**

**Institutes ^1^Renal Division, Department of Medicine，West China Hospital of Sichuan University; Kidney Research Institute; Chengdu 610041, China**

**^2^Renal Division, Department of Medicine，Affiliated Hospital of Chengdu University, Chengdu 610081, China**

**^3^Department of Biostatistics, School of Public Health, University of Michigan, Ann Arbor, MI, United States of America**

**Correspondence: Shen-Ju Gou**

**Renal Division, Department of Medicine，West China Hospital of Sichuan University; Kidney Research Institute; Chengdu 610041, China**

**Fax:** **+86-28-85423341**

**Telephone: +86-28-85164167**

**E-mail:** [**goushenju@163.com**](mailto:goushenju@163.com)

Supplementary Table S1 comparison of clinical features and laboratory findings according to different thyroid dysfunction identification in NS patients without steroid treatment.

|  | | Normal Thyroid | Subclinical hypothyroidism | Hypothyroidism | | Euthyroid sick syndrome | | | P^#^ |
| --- | --- | --- | --- | --- | --- | --- | --- | --- | --- |
|  | |  |  |  |  | Low T3 | Low T4 | Low T3T4 |  |
| Clinical characteristics | | |  |  | |  |  |  |  |
| N | 32 | | 42 | | 46 | 32 | 3 | 22 |  |
| Age (Year) | 42.25±14.37 | | 43.67±16.25 | | 37.02±16.44 | 45.63±15.85 | 40.67±17.01 | 40±14.3 | .223 |
| Duration of NS (Month) | 6.05±9.19 | | 5.61±8.72 | | 5.22±10 | 11.42±22.68 | 3.23±2.66 | 5.71±7.46 | .329 |
| PRO (g/24h) | 4.28±2.35 | | 7.26±4.38 | | 10.11±6.69* | 6.83±4.76 | 6.87±5.59 | 12.03±8.4* | .000 |
| PCR (g/mmol Cr) | 0.52±0.45 | | 0.91±0.5 | | 1.01±0.53* | 0.75±0.43 | 0.67±0.4 | 1.08±0.98* | .033 |
| ALB (g/L) | 28.2±5.89 | | 23.23±5.45* | | 19.11±4.29* | 24.85±4.16* | 23.93±6.43 | 19.14±3.65* | .000 |
| SCR (μmol/L) | 61.5[51.25,93.75] | | 71.15[54.75,88.975] | | 65.3[47.95,96.125] | 79.55[59.675,167] | 54[38,67] | 79.25[55.475,147.85] | 0.089 |
| UA (μmol/L) | 341.55±78.77 | | 372.98±106.92 | | 339.75±71.3 | 349.77±93.46 | 324.77±123.68 | 357.51±106.32 | .637 |
| TG (mmol/L) | 2.08±1.26 | | 2.41±1.21 | | 2.28±1.31 | 1.87±1.01 | 1.49±0.62 | 2.51±1.33 | .264 |
| TC (mmol/L) | 7.45±2.44 | | 8.13±3.16 | | 9.14±2.82 | 6.92±2.7 | 5.88±1.49 | 9.21±3.98 | .006 |
| LDL (mmol/L) | 4.92±1.98 | | 5.42±2.68 | | 6.31±2.48 | 4.39±2.28 | 3.69±1 | 6.48±3.72 | .007 |
| FT3 (pmol/L) | 4.54±0.67 | | 4.23±1.04* | | 2.82±0.68* | 3.41±2.66 | 4.22±0.41 | 2.45±0.75* | .000 |
| TT3 (nmol/L) | 1.72±0.26 | | 1.62±0.52 | | 1.13±0.21 | 1.23±0.42 | 2.04±0.1 | 1.21±0.32 | .001 |
| FT4 (pmol/L) | 15.5±2.1 | | 14.53±3.03* | | 10.76±1.97 | 14.45±2.25* | 11.21±1.12 | 10.19±1.3* | .000 |
| TT4 (nmol/L) | 89.03±11.33 | | 86.65±20.95 | | 50.62±14.88 | 85.99±28.8 | 83.51±0.2 | 53.36±10.56 | .000 |
| TSH (mU/L) | 3.19[2.2725,3.84] | | 6.21[4.985,7.035] | | 7.325[5.7175,9.2275] | 2.835[1.91,3.35] | 3.23[1.15,3.27] | 2.745[2.4075,3.21] | 0 |
| RT3 (nmol/L) | 0.32±0.15 | | 0.3±0.1 | | 0.21±0.07 | 0.24±0.06 | 0.25±0.03 | 0.3±0.09 | .058 |
| HB (g/L) | 143.84±27.42 | | 130.4±25.22 | | 138.2±20.86 | 121.09±29.34* | 135.33±7.02 | 129.95±28.84 | .012 |
| WBC (10^9/L) | 6.24±1.62 | | 7.13±2.37 | | 7.07±2.35 | 7.35±3.87 | 5.45±2.83 | 7.41±3.22 | .463 |
| PLT (10^9/L) | 195.03±64.64 | | 240.38±109.33 | | 242.04±70.05 | 212.53±78.14 | 209.33±19.86 | 245.55±85.86 | .097 |
| Renal biopsy (%) |  | |  | |  |  |  |  |  |
| MCD | 18.75% | | 21.95% | | 54.35% | 37.50% | 0.00% | 43.48% | 0.028 |
| MPGN | 0.00% | | 2.44% | | 0.00% | 0.00% | 0.00% | 8.70% |  |
| MsPGN | 0.00% | | 2.44% | | 0.00% | 6.25% | 0.00% | 4.35% |  |
| MN | 43.75% | | 41.46% | | 21.74% | 25.00% | 0.00% | 21.74% |  |
| FSGS | 6.25% | | 4.88% | | 2.17% | 0.00% | 100.00% | 4.35% |  |
| IgAN | 15.63% | | 12.20% | | 2.17% | 9.38% | 0.00% | 8.70% |  |
| Secondary NS | 15.63% | | 14.63% | | 19.57% | 21.88% | 0.00% | 8.70% |  |

Data are expressed as mean ± standard deviation, median and interquartile range, or percent frequency, as appropriate.

*MN* membranous nephropathy, *MCD* minimal-change disease, *FSGS* focal segmental glomerulosclerosis, *MPGN* membrane proliferative glomerulonephritis, *MsPGN* mesangial proliferative glomerulonephritis, *secondary* secondary reasons of nephrotic syndrome.

^#^ Comparison among groups. * P<0.05, Comparison versus normal thyroid group.

Supplementary Table S2. comparison of pathological types composition in different thyroid dysfunction subtypes of NS patients according to albumin level.

|  | Normal Thyroid | Subclinical hypothyroidism | Hypothyroidism | Euthyroid sick syndrome | | |  |  |  |  |  |  |  |  |  |  |
| --- | --- | --- | --- | --- | --- | --- | --- | --- | --- | --- | --- | --- | --- | --- | --- | --- |
|  |  |  |  | Low T3 | Low T4 | Low T3T4 | P# |  | MCD | MPGN | MsPGN | MN | FSGS | IgAN | Secondary | P# |
| ALB≤17 (g/L) |  |  |  |  |  |  |  |  |  |  |  |  |  |  |  |  |
| n | 2 | 9 | 28 | 6 | 0 | 15 |  |  | 39 | 1 | 0 | 9 | 2 | 4 | 5 |  |
| PRO (g/24h) | 3.81±0.13 | 10.56±5.1 | 14.56±9.2 | 6.35±3.15 |  | 16.14±11.5 | 0.11 |  | 14.93±9.69 | 7.17 |  | 11.97±6.58 | 3.89±0.1 | 14.49±13.89 | 6.03±3.84 | 0.32 |
| ALB (g/L) | 16.2±0.28 | 15.67±1.1 | 14.09±1.91 | 15.6±1 |  | 15.32±0.93 | 0.01 |  | 15.06±1.35 | 16.8 |  | 13.97±2.31 | 14.9±0.2 | 13.95±1.79 | 15.36±1.84 | 0.25 |
| Renal biopsy (%) |  |  |  |  |  |  |  |  |  |  |  |  |  |  |  |  |
| MCD | 100% | 55.60% | 60.70% | 83.30% |  | 66.70% | 0.47 |  |  |  |  |  |  |  |  |  |
| MPGN | 0 | 11.10% | 0 | 0 |  | 0 |  |  |  |  |  |  |  |  |  |  |
| MsPGN | 0 | 0 | 0 | 0 |  | 0 |  |  |  |  |  |  |  |  |  |  |
| MN | 0 | 22.20% | 14.30% | 0 |  | 20.00% |  |  |  |  |  |  |  |  |  |  |
| FSGS | 0 | 0 | 0 | 16.70% |  | 6.70% |  |  |  |  |  |  |  |  |  |  |
| IgAN | 0 | 1 | 10.70% | 0 |  | 0 |  |  |  |  |  |  |  |  |  |  |
| Secondary | 0 | 0 | 14.30% | 0 |  | 6.70% |  |  |  |  |  |  |  |  |  |  |
| 17 (g/L) <ALB≤20 (g/L) | |  |  |  |  |  |  |  |  |  |  |  |  |  |  |  |
| n | 4 | 6 | 20 | 7 | 2 | 13 |  |  | 24 | 3 | 1 | 10 | 0 | 1 | 13 |  |
| PRO (g/24h) | 5.86±3.59 | 7±4.81 | 5.49±2.76 | 7.05±6.1 | 4.54±2.06 | 9.03±5.13 | 0.11 |  | 4.91±3.23 | 8.03±0.59 | 12.38 | 7.4±5.49 |  | 4.45 | 8.41±4.43 | 0.12 |
| ALB (g/L) | 19.43±0.5 | 17.77±1.08 | 18.27±0.84 | 18.31±0.96 | 18.65±1.06 | 18.69±0.92 | 0.01 |  | 18.41±0.91 | 19.63±0.29 | 19.1 | 18.3±0.96 |  | 17.8 | 18.26±0.98 | 0.26 |
| Renal biopsy (%) |  |  |  |  |  |  |  |  |  |  |  |  |  |  |  |  |
| MCD | 50% | 33.33% | 65% | 42.86% | 0% | 30.77% | 0.05 |  |  |  |  |  |  |  |  |  |
| MPGN | 0% | 0% | 0% | 0% | 0% | 23.08% |  |  |  |  |  |  |  |  |  |  |
| MsPGN | 0% | 0% | 0% | 0% | 0% | 7.69% |  |  |  |  |  |  |  |  |  |  |
| MN | 0% | 16.67% | 20% | 0% | 100% | 23.08% |  |  |  |  |  |  |  |  |  |  |
| FSGS | 0% | 0% | 0% | 0% | 0% | 0% |  |  |  |  |  |  |  |  |  |  |
| IgAN | 0% | 16.67% | 0% | 0% | 0% | 0% |  |  |  |  |  |  |  |  |  |  |
| Secondary | 50% | 33.33% | 15% | 57.14% | 0% | 15.38% |  |  |  |  |  |  |  |  |  |  |
| 20 (g/L)<ALB≤30 (g/L) | |  |  |  |  |  |  |  |  |  |  |  |  |  |  |  |
| n | 28 | 36 | 35 | 42 | 2 | 17 |  |  | 37 | 2 | 6 | 65 | 6 | 15 | 29 | 0 |
| PRO (g/24h) | 4.16±2.11 | 6.34±3.79 | 7.92±5.13 | 7.48±5.02 | 12.72±0.17 | 7.69±4.92 | 0.01 |  | 6.47±4.48 | 3.23±0.13 | 6.6±6.77 | 6.91±4.47 | 8.48±5.55 | 6.14±3.43 | 7.25±4.85 | 0.84 |
| ALB (g/L) | 26.38±2.96 | 24.96±3.02 | 23.44±2.7 | 25.57±2.73 | 23.2±0.1 | 23.69±2.64 | 0.01 |  | 24.58±3.08 | 24.85±6.01 | 27.23±2.6 | 24.95±2.98 | 24.62±3.35 | 24±2.84 | 25.14±2.72 | 0.45 |
| Renal biopsy (%) |  |  |  |  |  |  |  |  |  |  |  |  |  |  |  |  |
| MCD | 17.86% | 13.89% | 31.43% | 28.57% | 50.00% | 17.65% | 0.43 |  |  |  |  |  |  |  |  |  |
| MPGN | 3.57% | 0.00% | 2.86% | 0.00% | 0.00% | 0.00% |  |  |  |  |  |  |  |  |  |  |
| MsPGN | 0.00% | 0.00% | 5.71% | 4.76% | 0.00% | 11.76% |  |  |  |  |  |  |  |  |  |  |
| MN | 53.57% | 58.33% | 28.57% | 28.57% | 50.00% | 35.29% |  |  |  |  |  |  |  |  |  |  |
| FSGS | 0.00% | 5.56% | 2.86% | 2.38% | 0.00% | 11.76% |  |  |  |  |  |  |  |  |  |  |
| IgAN | 10.71% | 8.33% | 5.71% | 11.90% | 0.00% | 11.76% |  |  |  |  |  |  |  |  |  |  |
| Secondary | 14.29% | 13.89% | 22.86% | 23.81% | 0.00% | 11.76% |  |  |  |  |  |  |  |  |  |  |
| 30 (g/L)<ALB |  |  |  |  |  |  |  |  |  |  |  |  |  |  |  |  |
| n | 23 | 10 | 0 | 11 | 1 | 0 |  |  | 10 | 1 | 2 | 18 | 4 | 6 | 4 |  |
| PRO (g/24h) | 3.56±2.7 | 3.81±3.22 |  | 3.82±3.53 | 1.76 |  | 0.93 |  | 2.69±3.34 | 0.88 | 4.98±4.84 | 3.29±1.99 | 2.62±1.62 | 4.42±3.19 | 7.1±4.1 | 0.17 |
| ALB (g/L) | 34.18±4.31 | 32.11±1.7 |  | 34.55±3.54 | 30.7 |  | 0.34 |  | 35.36±4.86 | 30.3 | 33.25±3.46 | 32.63±2.45 | 34.45±5.23 | 35.15±4.03 | 32.85±3.64 | 0.49 |
| Renal biopsy (%) |  |  |  |  |  |  |  |  |  |  |  |  |  |  |  |  |
| MCD | 30.43% | 10.00% |  | 18.18% | 0.00% |  | 0.97 |  |  |  |  |  |  |  |  |  |
| MPGN | 4.35% | 0.00% |  | 0.00% | 0.00% |  |  |  |  |  |  |  |  |  |  |  |
| MsPGN | 0.00% | 10.00% |  | 9.09% | 0.00% |  |  |  |  |  |  |  |  |  |  |  |
| MN | 34.78% | 50.00% |  | 36.36% | 100.00% |  |  |  |  |  |  |  |  |  |  |  |
| FSGS | 8.70% | 10.00% |  | 9.09% | 0.00% |  |  |  |  |  |  |  |  |  |  |  |
| IgAN | 13.04% | 10.00% |  | 18.18% | 0.00% |  |  |  |  |  |  |  |  |  |  |  |
| Secondary | 8.70% | 10.00% |  | 9.09% | 0.00% |  |  |  |  |  |  |  |  |  |  |  |

Data are expressed as mean ± standard deviation, median and interquartile range, or percent frequency, as appropriate.

Patients with different pathological types in different albumin level showed no significant differences in albumin and proteinuria.

*MN* membranous nephropathy, *MCD* minimal-change disease, *FSGS* focal segmental glomerulosclerosis, *MPGN* membrane proliferative glomerulonephritis, *MsPGN* mesangial proliferative glomerulonephritis, *secondary* secondary reasons of nephrotic syndrome.

^#^ Comparison among groups.

Supplementary Table S3. Clinical characteristics according to different pathological classifications.

|  | MCD | MPGN | MsPGN | MN | FSGS | IgAN | Secondary | P^#^ |
| --- | --- | --- | --- | --- | --- | --- | --- | --- |
| PRO (g/24h) | 8.79±7.88 | 7.55±5.37 | 7.07±6.43 | 6.75±5.02* | 4.41±4.51 | 7.29±6.91 | 7.44±4.41 | 0.29 |
| PCR (g/mmol Cr) | 0.91±0.52 | 1.22±0.74 | 0.61±0.27 | 0.75±0.6* | 0.4±0.21 | 0.84±0.73 | 0.82±0.54 | 0.25 |
| ALB (g/L) | 20.82±6.43 | 21.58±4.57 | 26.47±5.8* | 24.59±5.74* | 29.81±8.18* | 24.98±7.48* | 23.13±5.35* | <0.001 |
| SCR (μmol/L) | 62.7[53,83.18] | 76.6[63.00,181.00] | 84.3[72.55,182.15] | 66[51.50,83.70]* | 68[47.00,122.70] | 79.6[67.75,107.73]* | 91[57.10,122.00]* | <0.001 |
| TG (mmol/L) | 2.58±1.47 | 1.81±0.86 | 2.75±2.47 | 2.28±1.29 | 3.14±0.7 | 2.2±1.05 | 2.52±2.06 | 0.37 |
| TC (mmol/L) | 9.75±3.13 | 7.13±3.02* | 8.33±4.91 | 7.75±2.68* | 8.01±3.41 | 7.18±2.85* | 6.89±2.46* | <0.001 |
| LDL (mmol/L) | 6.59±2.78 | 4.68±2.4 | 5.63±3.95 | 4.83±2.29* | 5.17±3.2 | 4.6±2.22* | 4.41±1.99* | <0.001 |
| HB (g/L) | 145.69±18.97 | 125.9±26.23* | 111.14±33.84* | 132.98±20.37* | 141.14±24.15 | 135.09±36.52 | 116±22.39* | <0.001 |
| WBC (10^9/L) | 7.56±2.61 | 9.04±2.59 | 8.87±4.67 | 8±3.12 | 8.01±2.1 | 7.91±3.5 | 7.9±4.16 | 0.77 |
| PLT (10^9/L) | 245.96±77.98 | 223.8±87.35 | 196±106.92* | 216.91±75.11* | 243.29±181.79 | 214.77±85 | 211.26±104.04* | 0.14 |

Data are expressed as mean ± standard deviation (SD), median and interquartile range (IQR) as appropriate.

*MN* membranous nephropathy, *MCD* minimal-change disease, *FSGS* focal segmental glomerulosclerosis, *MPGN* membrane proliferative glomerulonephritis, *MsPGN* mesangial proliferative glomerulonephritis, *secondary* secondary reasons of nephrotic syndrome.

^#^ Comparison among groups. * P<0.05, Comparison versus MCD group.

Supplementary Table S4. The correlations between these clinical parameters using spearman correlation analysis.

|  | PRO  (g/24h) | ALB  (g/L) | SCR  (μmol/L) | TG  (mmol/L) | TC  (mmol/L) | LDL  (mmol/L) | HB  (g/L) | WBC  (10^9/L) | PLT  (10^9/L) | Pathological types | Hypertension | Diabetes | Pneumonia |
| --- | --- | --- | --- | --- | --- | --- | --- | --- | --- | --- | --- | --- | --- |
| PRO (g/24h) | 1.000 | -.444^**^ | .260^**^ | .188^**^ | .176^**^ | .192^**^ | -.019 | .072 | .106 | -.083 | .106 | .023 | .140^*^ |
| ALB (g/L) | -.444^**^ | 1.000 | .012 | -.205^**^ | -.463^**^ | -.489^**^ | -.075 | -.026 | -.262^**^ | .303^**^ | .180^**^ | .070 | -.152^**^ |
| SCR (μmol/L) | .260^**^ | .012 | 1.000 | -.036 | -.252^**^ | -.227^**^ | -.177^**^ | .075 | -.083 | .112 | .279^**^ | .069 | .137^*^ |
| TG (mmol/L) | .188^**^ | -.205^**^ | -.036 | 1.000 | .521^**^ | .473^**^ | .125^*^ | .095 | .114 | -.097 | .114 | -.058 | -.041 |
| TC (mmol/L) | .176^**^ | -.463^**^ | -.252^**^ | .521^**^ | 1.000 | .961^**^ | .373^**^ | .073 | .293^**^ | -.387^**^ | -.116^*^ | -.145^*^ | -.053 |
| LDL (mmol/L) | .192^**^ | -.489^**^ | -.227^**^ | .473^**^ | .961^**^ | 1.000 | .360^**^ | .030 | .317^**^ | -.369^**^ | -.094 | -.138^*^ | -.024 |
| HB (g/L) | -.019 | -.075 | -.177^**^ | .125^*^ | .373^**^ | .360^**^ | 1.000 | .254^**^ | .154^**^ | -.334^**^ | -.204^**^ | -.153^**^ | -.084 |
| WBC (10^9/L) | .072 | -.026 | .075 | .095 | .073 | .030 | .254^**^ | 1.000 | .378^**^ | .038 | .025 | -.012 | .042 |
| PLT (10^9/L) | .106 | -.262^**^ | -.083 | .114 | .293^**^ | .317^**^ | .154^**^ | .378^**^ | 1.000 | -.214^**^ | -.083 | .031 | .075 |
| Pathological types | -.083 | .303^**^ | .112 | -.097 | -.387^**^ | -.369^**^ | -.334^**^ | .038 | -.214^**^ | 1.000 | .170^**^ | .070 | -.049 |
| Hypertension | .106 | .180^**^ | .279^**^ | .114 | -.116^*^ | -.094 | -.204^**^ | .025 | -.083 | .170^**^ | 1.000 | .188^**^ | .043 |
| Diabetes | .023 | .070 | .069 | -.058 | -.145^*^ | -.138^*^ | -.153^**^ | -.012 | .031 | .070 | .188^**^ | 1.000 | -.048 |
| Pneumonia | .140^*^ | -.152^**^ | .137^*^ | -.041 | -.053 | -.024 | -.084 | .042 | .075 | -.049 | .043 | -.048 | 1.000 |

* P<0.05; ** P<0.01.
